# On Diseases of the Eye

**Published:** 1844-06-01

**Authors:** 


### RECORD OF MEDICAL SCIENCE.

We cannot deny ourselves the pleasure of laying before our readers the following remarks of the celebrated Velpeau, on a subject that interests every physician who regards the dignity of science and the honor of his profession. "*Specialties*" are coming into vogue here, not less than in the cities of Europe; and those who are looking that way, will do well to consider in what light the subject is regarded by those who are capable of appreciating such conduct, but who are above stooping to embrace it.—Ed.

### ON DISEASES OF THE EYE.

BY PROFESSOR VELPEAU.

*Introductory Lecture.*—These diseases are extremely frequent, and the truth of this assertion may be verified daily in the hospitals; but as many practitioners make mistakes, it may not be uninteresting nor useless to pass them in review; and, at the same time, I will add some further details to those which I gave in my lectures some years ago. The eye being one of the most important organs of the animal economy, naturally, practitioners in all ages have studied its diseases attentively, and placed them in a category separate from other disorders. Thus, we find the germ of this specialty in the writings of the Hebrews and Egyptians. Reasons have been urged for and against this tendency, but these being foreign to our subject, need not here be discussed. If we reflect for an instant on the organization of the eye, we shall easily conceive why it and its appendages (the eye-lids, vessels, nerves, muscles, and lachrymal ducts,) are so frequently the seat of disease; and being composed of elements differing essentially in their nature, are consequently susceptible of producing disorders, *sui generis*. For instance, do not the eyelids offer in their composition tissues very different from each other? Do we not find there integuments of a peculiar nature, cellular and muscular tissues, cartilages, glands, and eye-lashes, (these last themselves formed of various elements;) and on their inner surface a mucous membrane, with its follicles and *villosities*? What a diversity of elements! Again, if we examine the lachrymal organs, we find the puncta, ductus, and saccus lachrymalis, the ductus nasalis, and the glandula lachrymalis. And for the eye itself, the conjunctiva, the sclerotica, the cornea, the choroidea, the retina, the iris, the crystalline lens and its capsule, the aqueous humour, the vitreous humour, and the hyaloidea; all these contained in an organ not larger than a walnut. But these parts are not separated from each other; on the contrary, they cross, and are closely united to each other. Now, it is generally admitted that these several parts may be the seat of diverse lesions, for the conjunctiva may be affected by the various forms of inflammation, and it is the same with the other elements which compose the organ.

These diseases offer several peculiarities worthy of notice. 1°. They may have their seat in the surrounding, or the component parts, of the eye, and may, therefore, be divided into external and internal. In the former they are visible; and consequently, the diagnosis is easily established; whilst in the latter, the difficulty is greater, and errors far more frequent. 2°. The consecutive accidents are very different from what is observed in the diseases of other organs. Thus, in the different viscera, inflammation may take place, and terminate by resolution, even when pus has been formed. This is not the case in the eye; for though the patient may be perfectly well in other respects, still he may lose his sight from the opacity produced in certain parts of the eye by the previous affection. The eye may, therefore, be considered as a special organ, and this accounts for the tendency of certain practitioners to make it the object of a specialty in medicine, which is, I do not hesitate in saying, highly dangerous. Doubtless, the advancement of science, and the good of mankind were the "*primum mobile*" of these authors, and no one would think of raising his voice against so praiseworthy an intention. But there is another motive why in medicine men adopt a specialty, a motive which, while excusable in other professions, is *beneath* one whose object is so noble as ours. In a word, in adopting a specialty, the aim of the individual is to place himself in a position to gain money; whilst that of the physician is, or ought to be, the relief of his fellow creatures. In acting otherwise, our noble profession is assimilated to a mechanical art, in which the artisan seeks his own interest, not that of his fellow creatures. The medical profession can never be considered as a business. But what occurs, in general, when an individual creates a specialty? He reads all that has been written on the subject; after which the object of his studies becomes disagreeable, however pleasing it may have been at first; and then, either he chooses another subject, which is certainly the most honourable, or he endeavours to turn to profit the knowledge he has acquired, and in order to attain his end, he must employ the necessary means. Thus, he must endeavour to make the public believe that he knows more than his colleagues, like a manufacturer who publishes that his merchandise is the best. Concealing the cases in which he fails, he vaunts his successes, loses thus the scientific honesty and true courage of the savant, and the works which he publishes, though perhaps containing excellent and instructive matter, are no longer of use to the scientific man, who dare not adopt them for fear of being led into error. But "*specialists*" pretend that as they have passed their life in studying a given subject, they ought to know it better than those who study all the diseases which affect the human frame. This opinion has not only been received among the public, but has also been adopted by many members of the profession, and yet however correct it may appear, it can easily be proved erroneous, for there is no part of the human frame which can be separated from the rest with impunity. Can the eye be considered as foreign to the organism? On the contrary, is it not the model of all other systems, as we find in it all that is to be met with elsewhere? Is it necessary to give a proof that what interests this organ, interests the rest of the economy? Are there not diseases of the eye which are produced by a carious tooth, by certain conditions of the brain, the liver, the lungs? Now, if an oculist neglected studying the disorders of these parts, how can he cure those of the eye. Thus, whilst in all ages, specialties have fallen to the lot of men of mediocre talent, those whose endowments are greater, and whose intelligence is more highly developed, concentrate the activity of their mind, first on one subject, and then on another, without studying one to the exclusion of all others. But it would be hardly worth while occupying ourselves with this question, were it not that I wish to lay before you the tactics of these individuals. If unknown, they have a patient whom they wish to show you, a new mode of operating which they ask leave to try, not publicly, but in your presence only. If they are in vogue, we have quite a different affair. They, and they alone, know how to cure diseases of the eyes; an opinion shared by numbers of our brethren, who, by advising their patients to consult these persons, aid this *manœuvre*, which really ought not to be considered as forming part of the conduct of a conscientious practitioner. And now, as far as concerns oculists, let us examine the pretensions they have the courage to emit as to their having promoted the science. To whom are we indebted for the different operations performed on the eye? To Pott, Scarpa, and Dupuytren for that of couching; to Richter, Wenzel, Boyer, and Professor Roux for that by extraction. In England, we find among those who have particularly distinguished themselves, Cheselden, Guthrie, Travers; Mackenzie (of Glasgow,) whose treatise is so highly and so deservedly esteemed, is not an oculist; as to Bier, what he left, is, comparatively speaking, of minor importance. Thus, it is evident that we owe the four-fifths of what is really useful on diseases of the eyes, to surgeons, not to specialists. Have we not, from surgeons, numerous articles in the different "dictionaries" which of late years have been published in France? Have we not, in Boyer's treatise, a volume in which these disorders are described? Again, there are few clinical professors who do not daily study them, and I myself delivered, some years ago, a course of lectures on this subject, at la Pitié. These preliminary remarks are sufficient to show how necessary it is for every physician to study diseases of the eye.

Some oculists have asserted that it is possible to discover from the nature of the disease, the constitution of the patient; thus while one kind of ophthalmia is of a scrofulous nature, another is of an hemorrhoidal, an hepatic, a varioloid, &c. This idea is but the reproduction of that of Sauvages, who considered these diseases as of a peculiar nature, and recommended a method which teaches us to study a class of disorders as we would plants, and which I have long since combated, not only because I consider it erroneous, but likewise because I think it dangerous. There is another point, as yet very obscure, but which I hope to elucidate in these lectures, viz., that no disease exists to which the prefix of scrofulous, rheumatismal, and such like, ought to be attached. Let it not be supposed, however, that I deny the existence of a scrofulous or rheumatismal taint; what I mean shall be explained as I proceed. All that I will say to-day is that ophthalmia in scrofulous patients is modified, as when it affects persons of a sanguineous or nervous temparament. But in order to prove beyond a doubt how erroneous the opinion is, which attributes such and such a disease of the eye to such and such a constitution, we need only examine a scrofulous person attacked with pleuritis, pericarditis, or any other disorder, and see if we discover any material difference in the symptoms. Who would ever think of calling pleuritis in such cases scrofulous pleuritis? Doubtless we must not omit, in studying the affection, to examine of what constitution a patient is, but I think it impossible to discover it from the examination of the eye. This organ, as we have already stated, is composed of numerous tissues, which may be affected separately or conjointly. Now when we say such an individual presents a scrofulous ophthalmia, we do not indicate what tissue is affected. Such a doctrine would lead to the supposition that the disorder, inflammation for instance, does not differ according to the tissue, or that the knowledge of the precise seat is of no importance whatever.—*London Medical Times.*

